# Case report: 11-ketotestosterone may potentiate advanced bone age as seen in some cases of Wiedemann-Steiner Syndrome

**DOI:** 10.3389/fendo.2022.1004114

**Published:** 2022-10-03

**Authors:** Katherine Buchanan, Erin Greenup, Anna C. E. Hurst, Bhuvana Sunil, Ambika P. Ashraf

**Affiliations:** ^1^ University of Alabama at Birmingham Marnix E. Heersink School of Medicine, Birmingham, AL, United States; ^2^ Division of Pediatric Endocrinology, Department of Pediatrics, Orlando Health Arnold Palmer Hospital for Children, Orlando, FL, United States; ^3^ Department of Genetics, University of Alabama at Birmingham, Birmingham, AL, United States; ^4^ Division of Pediatric Endocrinology and Diabetes, Mary Bridge Children’s Hospital, Tacoma, WA, United States; ^5^ Divison of Pediatric Endocrinology and Diabetes, Department of Pediatrics, University of Alabama at Birmingham, Birmingham, AL, United States

**Keywords:** 11-Ketotestosterone, Advanced Bone Age, Wiedemann-Steiner Syndrome, adrenarche, adrenal steroidogenesis

## Abstract

**Context:**

Wiedemann-Steiner Syndrome (WSS) is a genetic disorder associated with an array of clinical phenotypes, including advanced bone age and short stature. 11-ketotestosterone (11KT) is a member of the group known as 11-oxygenated C19 androgens that are implicated in premature adrenarche.

**Case description:**

Case 1: The patient is a 3 year and 11-month-old female diagnosed with WSS due to deletion of *KMT2A* detected on CGH microarray. At two years and 11 months, imaging revealed an advanced bone age. We obtained an 11KT level on this patient. 11KT in case 1 was elevated at 26.3 ng/dL, while the normal reference range is 7.3-10.9 ng/dL and the reference interval for premature adrenarche is 12.3-22.9 ng/dL, The repeat 11KT at follow up (chronological age 4 years and 6 months) was still elevated at 33.8 ng/dL Case 2: A second child with WSS and a 5kb intragenic *KMT2A* deletion was evaluated at 11 months of age; his 11KT was 4.5 ng/dL.

**Conclusions:**

The elevated 11KT may indicate maturational changes related to increasing adrenal gland androgenic activation and may explain the advanced bone age seen in some patients with WSS. To our knowledge, this is the first case report that describes 11KT as a bioactive androgen potentially causing bone age advancement in WSS. Lack of elevation of 11KT in the second child who is an infant suggests increasing androgenic precursors and metabolites related to premature adrenarche may need to be longitudinally followed.

## Introduction

Wiedemann-Steiner Syndrome (WSS) is a rare autosomal dominant condition first described by Wiedemann et al. in 1989 and expanded by Steiner and Marques in 2000 (1–3). Common findings include intellectual disability, dysmorphic facial features, short stature, developmental delay, and hypertrichosis including hypertrichosis cubiti (4, 5). WSS is associated with mutations in the *KMT2A* gene which encodes a histone methyltransferase that has a regulatory role in gene expression (6).

A consistent finding in WSS is dysregulated bone age, including previous reports of both delayed and advanced bone age (7). Bone age is most often measured through radiographs of the left hand and wrist or knee and scored with standardized methods such as Greulich and Pyle (8). Several case reports and cohort studies have identified individuals with WSS who also display an advanced bone age (4, 7, 9, 10). Advanced bone age is defined as a bone age that is two or more standard deviations above chronological age (11).

In general, causes of advanced bone age include familial constitutional advancement, sex steroid exposure, obesity, precocious puberty, tumors, adrenal disease, hyperthyroidism, and overgrowth syndromes (8). The mechanism behind WSS and bone age advancement remains unclear (12).

The main sex steroids that are known to have an influence on bone age are estrogens and androgens (13), primarily androstenedione, testosterone and dehydroepiandrosterone (DHEA). During puberty, the adrenal gland contributes to androgen production in females (14). The adrenal gland also produces a unique set of 11-oxygenated 19-carbon steroids, which are emerging as clinic biomarkers of androgen excess (15), eventhough not currently used clinically in cases of androgen excess. It is not yet known how the 11-oxygenated 19-carbon steroids might contribute to premature adrenarche or accelerated bone age, however studies have demonstrated that these androgens are elevated in premature adrenarche, suggesting their putative role as bioactive androgens (16–18). One specific 11-oxyandrogen metabolite, 11-ketotestosterone (11KT) is notable as it displays similar androgenic activity to testosterone including activating the androgen receptor and is perhaps a clinically relevant potent agonist of the androgen receptor in humans (19).

Here, we report two patients with WSS due to deletion of *KMT2A*, one of which who presented with advanced bone age despite short stature and poor growth. This case report qualified for Institutional Review Board (IRB) exemption from the University of Alabama IRB.

## Case report

**Case 1**: The patient is a 3 year and 11-month-old, African American girl diagnosed with WSS at nine months of age through a comparative genomic hybridization (CGH) array. Her weight is 13.7 kg (12^th^ percentile), height is 97 cm (20^th^ percentile), and the body mass index (BMI) is 14.5 kg/m^2^ (26^th^ percentile). She was a full term baby born *via* vaginal delivery with no known antenatal or perinatal complications. Her birth weight was 2.98 kg (6^th^ percentile, z-score -1.50 SD) and birth length (0.2 percentile, Z score -2.76 SD) was 45.7 cm. Family history was notable for anemia in her brother and hypertension and diabetes mellitus in multiple relatives.

At 15 weeks of age, the patient was admitted to the hospital due to poor weight gain and failure to thrive (FTT). Hypotonia was noted, which was attributed to poor nutrition. Feeding therapy was initiated to address the apparent malnutrition. She was also found to have a moderate patent ductus arteriosus (PDA). The patient was discharged once feeding improved. The patient was admitted to the hospital at seven months of age for pneumonia refractory to outpatient treatment. During this stay, persistent feeding difficulties, lack of growth, developmental delay, and constipation were noticed. At nine months of age, the patient was found to have continued FTT with a weight of 5.44 kg (<1^st^ percentile) and height of 64.50 cm (1^st^ percentile). The PDA was closed at 10 months of age in a transcatheter procedure.

A CGH array was performed using the Agilent 4x180k aCGH + SNP array and revealed three DNA abnormalities in a peripheral blood specimen. The most relevant is 430kB deletion at 11q23.3 (117,965,223 and 118,394,787) (GRCh37/hg19), which encompasses 14 refSeq genes and includes *KMT2A*. Metaphase fluorescence *in situ* hybridization (FISH) analysis using the MLL (*KMT2A*) probe confirmed this deletion. Due to the inclusion of the *KMT2A* gene in this heterozygous loss-of-function pathogenic deletion, a diagnosis of WSS was made. This result includes the entirety of *KMT2A*, which is haploinsufficient, and therefore associated with a pathogenic loss-of-function, which is the mechanism of WSS. In addition, the microarray detected a variant of uncertain significance (VUS) 237kb deletion at 16p13.3 (6,943,310-7,180,460) [including *RBFOX1*, which may be associated with neurodevelopmental and neuropsychiatric disorders (19, 20)] and a 288kb duplication VUS at 2p16.3 (47,8909,992-48,179,514) which includes two OMIM genes – *MSH6* and *FBXO11*. Parental testing was not obtained due to cost.

**Figure 1:**
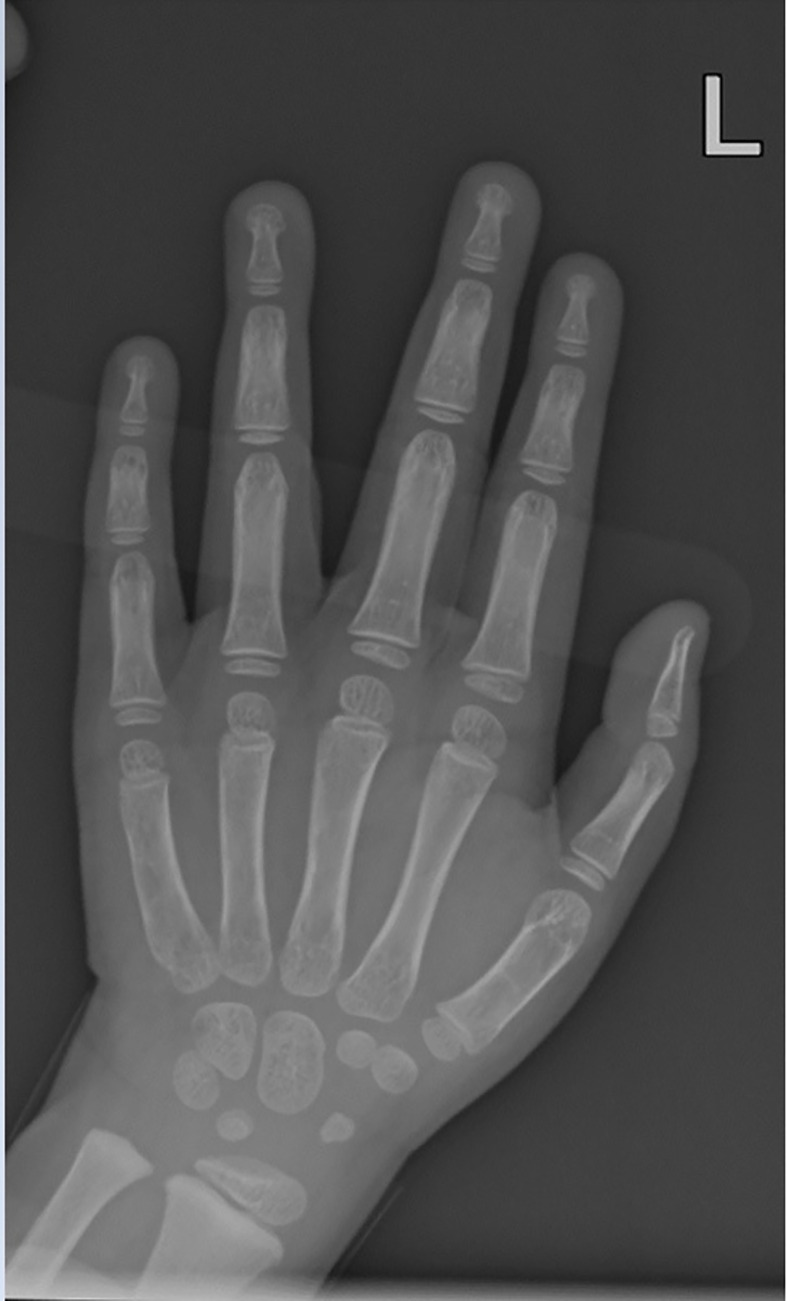
Bone age film for case 1, female child. The carpal bones were noted to be closest to five years while the phalangeal bones were found to be between four years and 2 months and five years using the Greulich and Pyle method, at chronological age two years and 11 months.

The patient started occupational, physical, and speech therapy following her diagnosis of WSS, and a gastrostomy tube was placed at 22 months of age to address the continued feeding difficulties. At two years and 11 months of age, the patient’s weight was 11.7 kg (8^th^ percentile), height 87.1 cm (6^th^ percentile), and BMI 15.4 kg/m^2^ (37^th^ percentile). The sex adjusted midparental target height was in the 90^th^ percentile. A bone age evaluation was performed due to her short stature, which revealed advanced skeletal maturity more than two standard deviations above the mean using the Atlas of Greulich and Pyle – **Figure 1**. The carpal bones were noted to be closest to five years while the phalangeal bones were found to be between four years and 2 months and five years. Causes of advanced bone age such as hyperthyroidism, central precocious puberty, and congenital adrenal hyperplasia were excluded. At this time, there were no clinical signs of premature adrenarche or thelarche. Her growth velocity was noted to be normal at 8 cm/year. A repeat bone age was 6 years and 10 months, while the chronological age was 3 years and 11 months.

Evaluation for congenital adrenal hyperplasia (CAH) revealed normal results: baseline 17-hydroxyprogesterone 37 ng/dL, androstenedione 34 ng/dl, testosterone < 10 ng/dL, 11-deoxycortisol 53 ng/dL, DHEA 198 ng/dL, progesterone 0.1 ng/mL, cortisol 15.5 mcg/dL, deoxycorticosterone <16 ng/dL, and 17-hydroxypregnenolone 213 ng/dL. At the age of 3 years and 5 months, a serum 11-ketotestosterone (11KT) level was measured by commercial laboratory (Esoterix, Inc., Calabasas Hills, CA, USA) using liquid chromatography with tandem mass spectrometric detection (LC-MS/MS). 11KT was 26.3 ng/dL, which is elevated when compared to published data on a reference population of girls aged 4-5 years (range 7.3-10.9 ng/dL) and girls with premature adrenarche age 4-7 years (range 12.3-22.9 ng/dL) (17). The repeat 11KT at follow-up (chronological age 4 year 6 months) was still elevated at 33.8 ng/dL.

The patient continues to receive care at the same institution and has planned follow-up appointments.

**Case 2**: During the evaluation of Patient 1, a second child was identified at our institution with WSS due to an intragenic deletion in *KMT2A*. We reviewed his medical records as an early assessment of a patient with a possible similar risk profile to help to determine at what age the lab values or image abnormalities may begin to show differences. Case 2 is an 11-month-old male with dysphagia, hip dysplasia, decreased height for age (69 cm at 11 months with Z-score -3.2), and characteristic facial features of WSS. Genome sequencing revealed a *de novo* 5kb deletion of part of exon 27 and all of exon 28 in *KMT2A* (chr11:118503405-118508421 (GRCh38). Deletions are a rare mechanism of WSS. A baseline 11-ketotestosterone level was obtained (4.5 ng/dL). Bone age evaluation is not reliable in this patient as he is less than two years of age.

## Discussion

Herein, we report the case of a patient with WSS with advanced bone age, concomitant with elevated 11KT, suggesting a possibility that 11-oxygenated C19 androgens could account for bone age advancement in some children with WSS. 11KT is postulated to be a dominant circulating bioactive androgen during normal and premature adrenarche (18). Following its secretion, 11KT can display androgenic effects by binding the androgen receptor (14). The 11-oxygenated steroids are primarily derived from adrenal tissue (16). These androgens, which have emerged as potential markers of adrenarche and conditions of androgen excess, are found to be particularly important in women and children (16). The potent androgenic 11-oxygenated steroids such as 11KT can bind and activate the human androgen receptor similar to testosterone and dihydrotestosterone (16). Due to this similar activity, it is therefore important to consider and measure these other androgens as they are clinically important especially in cases of androgen excess (20).

WSS syndrome affects males and females equally and is present among different populations with an overall estimated prevalence of <1 in 1,000,000 (21, 22). The genetic basis of WSS is a mutation in the *KMT2A* (also known as *MLL*) gene on chromosome 11q23, thought to result in haploinsufficiency of the gene and lead to the characteristic findings of the syndrome (3, 23). As *KMT2A* is expressed in a wide variety of tissues, WSS results in abnormalities in multiple body systems along with a wide variety of clinical findings (3). As the use of whole exome sequencing has increased, more individuals with WSS have been diagnosed, expanding the phenotypic spectrum of this condition. The molecular basis of most cases of WSS are single nucleotide variants (frameshifts, missense, nonsense, and splice-site variants) (10). Deletions are a rare mechanism which have only been reported once prior, in a female with advanced bone age and a deletion of exons 2-10 of *KMT2A* (12). This report adds an additional two cases of intragenic or whole-gene deletions of *KMT2A*, with one patient also having advanced bone age and a second patient whose evaluation did not reveal advanced bone age. However, this may be due to young age (11 months), so we will continue to monitor him for abnormal skeletal maturation.

Further research needs to be done to understand if elevated 11KT is seen in other patients with WSS, at what age exaggerated adrenal steroidogenesis is manifested, the potential reasons for this, and if there is an association between large multi-exonic deletions in *KMT2A* and advanced bone age. In case 1, the elevated 11KT marks an excess level of androgens that we hypothesize influenced bone maturation and led to the advanced bone age seen in this patient. Although our patient did not have significant clinical characteristics for premature adrenarche, her 11KT values were very similar (albeit slightly higher) to those reference intervals provided by the reference laboratory for premature adrenarche, while being 2.5 times higher than age-appropriate values.

The elevated 11KT level may indicate premature adrenarche due to the developmental maturation of the adrenal gland. The elevated 11KT in case 1 was not marked enough to suspect an adrenal tumor or warrant adrenal imaging. Moreover, other endocrinopathies, such as congenital adrenal hyperplasia, were excluded. The lack of clinical phenotype of premature adrenarche while having elevated urinary C_19_ steroids from age 3 years has been reported previously (24). We postulate that in some cases of WSS, this process of early adrenal maturation could be exponentially pronounced, accounting for elevated serum 11KT and accelerated bone age advancement.

Low birthweight and small for gestational age are known risk factors from metabolic programming for premature adrenarche (25). Early elevated 11KT may reflect the premature onset of adrenal gland maturation. The recent elucidation of conditions such as premature adrenarche and polycystic ovary syndrome manifesting with elevated 11KT (26), supports the notion of a mechanistic role for 11KT in premature adrenarche and conditions causing androgen excess. It is unclear if the increasing 11KT has a specific androgenic affinity for peripheral tissues such as bone and promotes skeletal maturation.

Genetic studies and mutational analysis indicate that the KMT family of histone lysine methylation-regulating enzymes interacts with the androgen nuclear receptor during hormone signaling (27). Interestingly, a genome-wide association study (GWAS) had shown that *KMT2A* can reportedly regulate steroidogenesis in animals (28). Perhaps the activity of the *KMT2A* gene might explain the abnormal steroidogenesis in WSS, accounting for the clinical phenotype of advanced bone age and ultimately short stature. Hence, it is possible that other factors related to *KMT2A* may play a role in bone age maturation in some children with this genetic diagnosis. This case expands the association of WSS and advanced bone age and suggests an association between 11KT and advanced bone age in patients with WSS. To our knowledge, this is the first case that suggests a role of 11KT in bone age advancement in a patient with WSS. The patient’s microdeletion does involve 14 total RefSeq genes, but the only one which has evidence for haploinsufficiency in the dominant state is *KMT2A*; others are not candidates for endocrinopathies (*MPZL2, UBE4A, CD3E, CD3G, CD3D, TMPRSS4, SCN4B, SCN2B, LOC100131526, ATP5MG, JAML, LOC101929089, MPZL3*), although this possibility cannot be excluded. Of note, the only prior reported patient with a deletion of *KMT2A* and advanced bone age had a deletion encompassing exons 2-10 (12). This more narrow deletion in a patient with a similar endocrinological phenotype gives some consistency that the *KMT2A* deletion is contributing to the advanced bone age and not another gene in our patient’s microdeletion.

Our second patient with WSS due to *KMT2A* intragenic deletion had an unremarkable 11KT, but it is unclear if this was because he is still an infant at the time of testing, an age at which adrenal steroidogenesis may not have been stimulated. We thought the inclusion of this patient with WSS helped to underscore the possibility that adrenal steroidogenesis in patients with WSS might occur at different ages (similar to variations in adrenarchal age in normal children). We will continue to follow this as he ages. His bone age could not be accurately evaluated due to his young age. In a recent cohort of 104 patients with WSS (10), none had a multi-exonic or whole-gene deletion as in our patients, who both had a clinical diagnosis of WSS. Therefore, these patients provide additional evidence that multiexonic deletions are a mechanism for loss-of-function and the WSS phenotype, which had previously only been described in the report by Mendelsohn, B.A., et al. (12). Also, in Sheppard, S.E., et al. (10),, bone ages were normal in 55.2%, advanced in 24.1% and delayed in 13.8% of 29 patients with WSS. Further studies are needed to see if advanced bone age is a major feature of WSS and if there is a genotype-phenotype correlation with the mechanism of *KMT2A* variation and endocrinological characteristics.

We recognize that our novel observation was limited to positive findings in one patient, and hence, this observation may not be generalizable. While the elevated 11KT cannot be definitively considered as the cause for advanced bone age in this case, with what is known about premature adrenarche, the elevated 11KT levels could possibly be responsible for the skeletal maturation seen in this patient. Systematic longitudinal studies in children with WSS are warranted to address the apparent link between premature adrenarche, advanced bone age, and the pathophysiologic role of elevated 11KT, and to ensure reproducibility. We also acknowledge that the reference ranges for 11-oxygenated androgens in pediatric populations have been limited to two publications based on small sample sizes (17, 18), and further studies are needed to validate age and sex-specific reference intervals. We do not have a DHEAS measurement in Case 1, however the DHEA concentration of Case 1 was normal. We also do not have any information on the diurnal variation of the 11KT production and the estradiol concentrations.

Traditionally, aromatization of androgens to estrogens is postulated to contribute to the advancement of bone age in patients with precocious puberty. However, at this time, it is not known whether 11KT is aromatizable. Another possibility is that other androgens, specifically 11-oxygenated androgens, could be produced from the adrenal gland and may cause advanced bone age, which is not a known association at the time of this writing. Serum androgen concentrations may also not correlate completely with bone age advancement at the epiphyseal level. While this report serves as a hypothesis-generating concept, more patients with diagnosed WSS need to be studied to generalize this finding.

## Conclusion

This case report highlights the potential role of elevated levels of 11KT in the clinical phenotype of elevated bone age observed in some patients with WSS. Further research needs to be done to elucidate the mechanism behind the elevation of 11KT in WSS and continue to expand the clinical phenotype of this syndrome. In addition, we present multi-exonic deletions as a mechanism of WSS; more studies are needed to determine if there is a genotype-phenotype correlation between large *KMT2A* deletions and bone age acceleration.

## Data availability statement

The original contributions presented in the study are included in the article/supplementary material. Further inquiries can be directed to the corresponding author.

## Ethics statement

Ethical review and approval was not required for the study on human participants in accordance with the local legislation and institutional requirements. Written informed consent to participate in this study was provided by the participants’ legal guardian/next of kin.

## Author contributions

KB performed outside research and literature review as well as patient chart review, authored first draft of manuscript, and edited subsequent drafts. EG and BS edited manuscript drafts. AH provided genetics perspective as well as edited drafts and contributed to writing. AA edited multiple drafts of manuscript, contributed to writing, helped with clinical context, and helped oversee manuscript development. All authors contributed to the article and approved the submitted version.

## Conflict of interest

The authors declare that the research was conducted in the absence of any commercial or financial relationships that could be construed as a potential conflict of interest.

## Publisher’s note

All claims expressed in this article are solely those of the authors and do not necessarily represent those of their affiliated organizations, or those of the publisher, the editors and the reviewers. Any product that may be evaluated in this article, or claim that may be made by its manufacturer, is not guaranteed or endorsed by the publisher.
